# Skinfolds Measurement Protocols and Standards: A Narrative Review

**DOI:** 10.1016/j.advnut.2026.100662

**Published:** 2026-05-26

**Authors:** Joaquim H Cintra, Jarson P Costa-Pereira, Wagner L Ripka, Maria I Freire-Correia, Filipe A Jesus, Analiza M Silva, Luís B Sardinha, Timothy G Lohman, Steven B Heymsfield

**Affiliations:** 1 Postgraduate Program in Translational Medicine, Department of Endocrinology, Federal University of Ceará, Fortaleza, Brazil; 2 Postgraduate Program in Nutrition, Department of Nutrition, Federal University of Pernambuco, Recife, Brazil; 3 Graduation Program in Biomedical Engineering, Federal University of Technology – Paraná, Curitiba, Brazil; 4 University of Fortaleza, Fortaleza, Brazil; 5 Exercise and Health Laboratory, Interdisciplinary Center for the Study of Human Performance, Faculdade Motricidade Humana, Universidade de Lisboa, Cruz-Quebrada, Portugal; 6 Atlântica, Fábrica da Pólvora de Barcarena, Instituto Universitário, Barcarena, Portugal; 7 Department of Movement Sciences and Sports Training, School of Sport Sciences, The University of Jordan, Amman, Jordan; 8 Emeritus Faculty, University of Arizona, Tucson, AZ, United States; 9 Pennington Biomedical Research Center, Baton Rouge, LA, United States

**Keywords:** body composition, anthropometry, skinfold thickness, protocols, reference standards

## Abstract

Skinfold thickness assessment is one of the most widely used anthropometric methods for estimating subcutaneous adipose tissue and overall body adiposity in clinical, epidemiological, and sports settings. Despite its long-standing application, substantial heterogeneity persists across measurement protocols and standardization systems, potentially affecting reproducibility and comparability across studies. This narrative review aims to critically synthesize the historical, conceptual, methodological, and technical development of the main skinfold measurement protocols and standards, systematically compare their characteristics, and examine their methodological and practical implications for research and clinical applications. A targeted bibliographic synthesis of classic textbooks, consensus manuals, and original peer-reviewed studies on surface anthropometry was conducted. Seventeen anatomical skinfold sites and 31 distinct technical descriptions were identified, including 22 site-selection protocols and 9 measurement standards. Five established standards were comparatively analyzed, with emphasis on anatomical landmarking, fold orientation, spatial pinching procedures, and temporal reading control. Similarities were observed in general technical principles, including right-side assessment, perpendicular caliper application, controlled reading time, and maintenance of fold elevation. However, persistent divergences were identified in anatomical definitions, fold orientation, landmark precision, spatial positioning of pinch and caliper jaws, and recommended reading time, with greater heterogeneity at trunk sites than limb sites. Although the reviewed protocols and standards were developed for broad application across age groups, this review primarily reflected their use in adolescents and adults, as skinfold assessment in infants and young children involves additional considerations related to growth dynamics and rapid changes in adipose tissue. Skinfold measurement protocols and standards are not interchangeable and reflect pluralistic, complementary international initiatives rather than a single institutional standardization process.

**Statement of Significance**

This review critically synthesizes the main skinfold measurement protocols and standards. It highlights persistent anatomical and procedural differences that may affect measurement reproducibility, comparability across studies, and the clinical interpretation of body composition estimates.

## Introduction

Before the widespread adoption of standardized measurement systems, anthropometric units derived from the human body were commonly used to quantify length and distance [1]. This historical context highlights a fundamental principle of anthropometric and clinical measurement: without rigorous standardization, reproducibility is compromised, and comparisons across individuals, populations, and studies become unreliable [2]. In response, systematic efforts to standardize anthropometric methods and develop specialized instruments progressively improved the rigor of body size, shape, proportion, and composition assessment. These advances enabled cross-population comparisons, study replication, and the cumulative development of research in health, clinical, and sports sciences [1,2], including the consolidation of skinfold measurements for assessing subcutaneous adiposity.

Several authors proposed structured procedures to standardize anatomical sites, reference landmarks, and measurement techniques. Weiner and Lourie [3], Behnke and Wilmore [4], Ross and Marfell-Jones [5], Lohman et al. [6], and later Esparza-Ros et al. [7] developed frameworks that became widely adopted by researchers, anthropometrists, and clinicians. Although developed in distinct institutional and historical contexts, these works collectively established the operational foundations of contemporary anthropometric practice by integrating principles intended to ensure uniformity, reliability, and consistency in body assessment [8].

As knowledge of body composition expanded, inconsistencies in terminology and conceptual models became increasingly evident. In 1992, Wang et al. [9] organized body composition into 5 levels: *atomic*, *molecular*, *cellular*, *tissue-system*, and *whole-body*. Among these, the molecular level (e.g., fat mass and fat-free mass) and the tissue-system level (e.g., adipose tissue, skeletal muscle tissue, and bone tissue) are most directly aligned with anthropometric assessment, as they correspond to compartments and structures that can be indirectly inferred through standardized somatic measurements. Classical anthropometric models, particularly those applied in vivo, traditionally partitioned steady-state body mass into 2 components based on these measurement principles [8]. Initially descriptive and morphological, such models were progressively incorporated into applications related to growth, performance, and health [2]. The emergence of kinanthropometry in the 1970s further consolidated anthropometric assessment as a distinct scientific field, integrating biological, functional, and environmental perspectives within a unified framework [10].

Within this established tradition, the skinfold technique evolved into a structured field-based procedure grounded in standardized anthropometric principles. This approach covers 2 interrelated methodological dimensions: the *protocols* defining the anatomical sites and their analytical combination, and the operational *standards* guiding measurement process, including site identification and landmarking, skinfold caliper specifications and calibration, pinch technique, observer training, and standardized timing before dial reading. Despite its practicality, portability, and low cost [8], these procedural components remain heterogeneous across published protocols and standards. Such variability may increase technical measurement error and limit comparability between studies and populations [7,8,11,12].

This narrative review aims to critically synthesize the conceptual, practical, historical, methodological, and technical development of the main skinfold measurement protocols and standards, systematically compare their characteristics, and examine their methodological and practical implications for research and clinical applications. Although the protocols and standards examined in this review were developed to be widely applicable across different age ranges, the present analysis primarily reflects their methodological consolidation in adolescent and adult populations. In infants and young children, skinfold assessment is influenced by rapid growth and dynamic changes in adipose tissue, which may require specific interpretive frameworks [11]. This review will be structured in 3 main sections: *1*) practical implications of skinfold measurement, *2*) historical development of protocols and standards, and *3*) a technical and methodological synthesis of their characteristics and applications.

## Development

### Skinfold measurement: practical implications

Skinfold thickness is defined as a double layer of skin and underlying subcutaneous adipose tissue and is commonly used as the primary anthropometric property to quantify local, regional, or total body adiposity in clinical, epidemiological, and sports sciences, owing to its practicality, low cost, and applicability in field settings [8]. Growing evidence supports the idea that when performed by trained anthropometrists, these measures show high intrarater and interrater reliability, provided that standardized anatomical sites, appropriate calipers, and uniform protocols are used [13]. They are indicated for monitoring changes in subcutaneous adiposity over time, including in nutritional assessment, considering that they are generally less sensitive to acute physiological variations related to the menstrual cycle, food intake, hydration status, and physical activity [8,13].

Although the skinfold technique is frequently used to estimate fat mass or adipose tissue mass through specific mathematical models, variability across regression equations, derivation samples, and measurement protocols limits the validity of skinfold measurements, especially in heterogeneous populations [8,13]. Different equations may perform differently across age groups, sexes, ethnicities, and clinical conditions, reducing their generalizability. Exceptions to these observations include population-specific skinfold-based equations that have been cross-validated [8,14]. For these reasons, several authors recommend assessment based on the sum of skinfold thicknesses, particularly at 6 or 8 anatomical sites, together with reference percentile curves as viable alternatives with greater methodological robustness [8,14]. This approach may avoid many of the assumptions embedded in predictive regression equations, allowing a more direct characterization of subcutaneous adiposity. Moreover, practical, financial, logistical, and biological constraints limit the use of reference methods, such as MRI, computed tomography, dual-energy X-ray absorptiometry, air displacement plethysmography, deuterium dilution, and ultrasound, especially under uncontrolled or field conditions [13]. In this context, skinfold-based assessments remain a reliable, accessible, and clinically relevant alternative [8,13,14].

Nevertheless, surface anthropometric variables, such as girths and skinfolds, incorporate multiple tissue components, and their individual contributions to the record values are not always clearly distinguishable. Skinfold thicknesses are predominantly composed of superficial subcutaneous adipose tissue [15]. At specific anatomical sites and adiposity patterns, it may also include deep adipose tissue [16]. Skin thickness and subcutaneous tissue compressibility can vary between individuals and are primarily influenced by sex, age, and anatomical site. These sources of variability potentially contribute to random measurement errors [15]. Under conditions of high adiposity, these factors can also introduce systematic bias due to inherent technical limitations in pinching, such as difficulty in keeping tissue layers parallel, compromising measurement accuracy.

Comparative studies have examined several technical determinants of skinfold thickness measurement [12,17,18]. Ruiz et al. [17] recommended applying the caliper jaws at an intermediate depth. Hume and Marfell-Jones [18] highlighted the importance of accurate site location. Vaquero-Cristóbal et al. [19] observed that most skinfold thicknesses reach minimum and stable values after ∼3 s under constant pressure. Finally, Cintra et al. [12] recommended that the target site be palpated beforehand to allow familiarization with the morphology and viscoelastic behavior of the skin–subcutaneous adipose tissue complex, followed by the determination and marking of the optimal pinch size. They further reported that trunk skinfolds generally require a pinch size ≥6 cm, whereas limb skinfolds (except the mid-thigh) typically require a pinch size ≤6 cm [12]. The thumb and index finger are drawn together to firmly grasp the fold. The amount to be pinched should be proportional to the tissue thickness at the site and minimally sufficient to form a skinfold with approximately parallel sides [6,12]. As an additional practical parameter, the anthropometrist should monitor the surrounding skin outside the fold margins, as excessive visible tension or stretching of this adjacent tissue may indicate that the pinch size exceeds the optimal range. In such cases, the grasp should be slightly reduced to avoid unnecessary tissue traction and to ensure that only the intended skin and subcutaneous adipose tissue are incorporated into the fold [12].

The downscale pressure applied by the skinfold caliper can displace extracellular fluid and allow adipose lobules to shift toward regions of lower pressure, dynamically altering tissue composition during measurement. This effect can be particularly pronounced in thick skinfolds with lower connective tissue content [12,14]. Consequently, these factors increase intrarater technical error measurement and reduce the sensitivity to detect small changes in subcutaneous adiposity over time [8]. The physical-mechanical configuration of skinfold calipers also has important methodological and practical implications.

**FIGURE 1:**
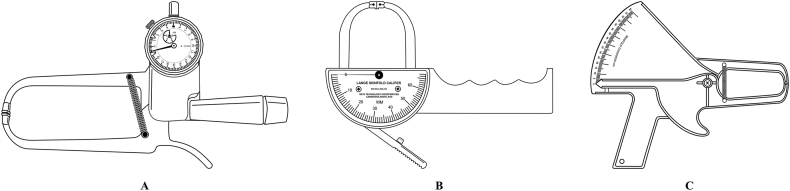
Cintra classification. (A) Original skinfold caliper type A: Harpenden. (B) Original skinfold caliper type B: Lange. (C) Original skinfold caliper type C: Slim Guide.

Differences in jaw surface area and spring force between calipers can influence tissue compression, fluid displacement, and skinfold stability [20]. Skinfold calipers that apply nonuniform or excessive pressure may exacerbate tissue deformation, particularly in thicker skinfolds. Consequently, the indiscriminate use of skinfold calipers with distinct mechanical behaviors may compromise measurement reliability, limit comparability across studies, and introduce systematic bias into skinfold-based body composition assessment [20]. In response to these methodological limitations and the lack of standardization in instrument selection, a new classification model was proposed in 2025. This classification systematically organizes skinfold calipers into 3 categories (original, generic, and hybrid) and 3 configurations (type A, type B, and type C) (Figure 1), based on their defining physical and mechanical properties and characteristics [21]. By shifting the focus from historically arbitrary or commercially motivated distinctions to objectively measurable attributes, such as lever class, jaw surface area, and spring force, this model establishes a robust methodological basis for instrument selection. Consequently, it increases transparency in reporting, facilitates comparability across studies, and contributes to greater methodological rigor and standardization in the assessment of skinfold measurements [20,21].

### Skinfold measurement: historical perspective on protocols and standards

Early skinfold thickness measurement practices were characterized by substantial procedural heterogeneity, which motivated successive international efforts toward methodological convergence during the 20th century [8,10]. Foundational textbooks and consensus initiatives played a key role in formalizing measurement frameworks and conventions, establishing the basis for the subsequent standardization of soft-tissue measurements, including skinfold thickness assessment.

Anthropologists and archaeologists, drawing on a long-standing tradition in anthropometry, particularly in skeletal measurements, played a central role in early efforts toward methodological standardization. A landmark milestone was the Frankfurt Agreement (1888), established during the 10th International Congress of Anthropology and Prehistoric Archaeology, which formally adopted foundational conventions for anthropometric measurements and provided a common anatomical reference framework that enhanced comparability across studies [3,10]. Notably, the 13th International Congress of Prehistory, Anthropology and Archaeology, held in Monaco in 1906, established the first formal agreements. The 14th International Congress of Anthropology and Archaeology, held in Geneva in 1912, later complemented these agreements. Although primarily institutional in nature, these initiatives established key conceptual and methodological foundations that were subsequently incorporated into anthropometric standardization. In 1914, Rudolph Martin formalized anthropometric methods in his textbook “*Lehrbuch der Anthropologie*,” with subsequent revisions until the late 1950s. The German school dominated anthropometry during the first half of the 20th century, and its influence spread to the United Kingdom and was later incorporated into research in sports science and sports medicine in North America [3,10].

Early investigations provided the empirical basis for measuring skinfold thickness. Richer [22] highlighted subcutaneous adiposity in body shape, whereas Oeder [23] and Batkin [24] extended these seminal observations to children and adults, proposing adipose thickness as a nutritional marker in clinical contexts. On the basis of these findings, Matiegka [25] introduced the first structured attempt to quantify body composition by separating different tissue compartments. However, Matiegka’s approach remained more theoretical than clinically formalized. It reflected the exploratory nature of early anthropometry, marked by inconsistent measurement choices and limited reproducibility and comparability across studies [8]. Terhedebrügge [26] later proposed systematic procedures to evaluate the relevance of 66 skinfold sites in describing body surface adipose patterns and distribution. This work represents an initial effort to establish more consistent protocols [27]. Similarly, Edwards [28] conducted an extensive investigation in which he measured 53 distinct skinfold sites in 138 women and emphasized regional variability in subcutaneous adiposity. These studies established the conceptual and practical foundations that directly informed subsequent methodological refinements in skinfold thickness measurement.

The study by Brozek and Keys [29] marked a crucial conceptual transition from predominantly descriptive anthropometry to a more analytical approach. By examining normative patterns and systematic relationships among anthropometric markers, the authors emphasized the importance of consistent measurement sites, rigorous operational definitions, and measurement reproducibility. Importantly, rather than treating skinfold thickness solely as a direct anatomical descriptor, they conceptualized it as a quantitative variable reflecting physiological adiposity. This methodological shift laid out the conceptual groundwork for later protocol development, although it largely preceded the broader methodological expansion and formal standardization efforts that emerged in subsequent decades.

From the 1950s onward, researchers expanded the application of the skinfold thickness technique by introducing specific measurement protocols and clearly defined anatomical sites [[27], [28], [29], [30], [31], [32], [33], [34], [35], [36], [37], [38], [39], [40], [41], [42], [43], [44], [45], [46], [47], [48], [49], [50], [51]], which were subsequently used to develop and validate regression equations for estimating body fat percentage based on densitometric methods and related assumptions [[52], [53], [54]]. When analyzed retrospectively, this postwar expansion reveals 2 important bibliographical approaches that structured the development of measurement protocols and standards before formal international institutionalization. A predominantly European approach, rooted in physical anthropology and human biology, emphasized the descriptive and morphological characterization of subcutaneous adiposity based on skinfold thickness [38]. Original studies, such as those by Hammond [32] and Tanner and Whitehouse [38], established reference values and measurement procedures for pediatric populations, reflecting an early interest in growth and development. These contributions were subsequently incorporated, either directly or indirectly, into anthropometric frameworks and standardization systems that became widely applied across all age ranges.

In parallel, an approach developed primarily in North America adopted a more applied orientation toward nutrition, health, and sports science, prioritizing analytical models, densitometric validation, and the early formalization of operational procedures for body composition assessment, as exemplified by Keys and Brozek [27] and Behnke and Wilmore [4]. Despite the increasing formalization of protocols, empirical evidence indicated that standardization alone was insufficient to ensure measurement comparability across examiners. Lohman et al. [55] found that individuals who applied standardized procedures but were not jointly trained exhibited examiner–caliper interactions that compromised measurement consistency and limited generalization. In response to these methodological challenges, international institutional initiatives aimed at anthropometric standardization were progressively consolidated throughout the 1970s and 1980s.

On 11 September, 1978, a Research Committee appointed by the *International Council of Sport Science and Physical Education (ICSSPE)*, meeting in Brasília, Brazil, established the *International Working Group on Kinanthropometry (IWGK)*. This working group aimed to promote and improve research related to kinanthropometry [56]. The IWGK’s anthropometric standards were subsequently published in 1982 by Ross and Marfell-Jones [5] as a chapter in the classic textbook “*Physiological Tests of the High-Performance Athlete*,” thus establishing an important methodological reference for the field. Subsequently, in 1985, an independent Anthropometric Standardization Conference was held in Airlie, Virginia, United States, with the support of the *NIH*. At this meeting, led by Timothy Lohman and Alex Roche, 50 experts from physical anthropology, epidemiology, human biology, exercise science, medicine, and nutrition met to discuss anthropometric standardization. At this conference, a consensus document was adopted that gave rise to the *Anthropometric Standardization Reference Manual (ASRM)* [6], which standardizes 40 anthropometric variables, later edited by Timothy Lohman, Alex Roche and Reynaldo Martorell, and published in 1988 [6].

Also in 1985, the IWGK was deactivated because of external administrative reasons. Consequently, the IWGK members reorganized themselves institutionally and, on 20 July, 1986, led by Jan Borms, Gaston Beunen, and Jim Day at a conference held at Jordanhill College of Education in Glasgow, Scotland, international delegates from 34 countries agreed to officially found the *International Society for the Advancement of Kinanthropometry (ISAK*) [10,56]. Furthermore, it was decided to integrate the ICSSPE as an official committee, and a constitution was established specifying 9 members on an Executive Council. Subsequently, although not institutionally adopted as official standards, the measurement procedures described in book chapters by Ross and Marfell-Jones [5,57] were widely used by ISAK members. Subsequently, in 1996, based on the *Australian Sports Commission’s Laboratory Standards Assistance Scheme*, ISAK approved the *International Anthropometry Accreditation Scheme* [56]. The anthropometric measurement technical procedures described in the textbook *Anthropometrica*, edited by Norton and Olds [58], were institutionally incorporated by ISAK to develop the *International Standards for Anthropometric Assessment*. These standards were formally published in 2001 and have since been periodically revised and updated [[59], [60], [61]]. The most recent edition, published in 2019 [7], standardizes 43 anthropometric variables organized into 2 technical profiles: restricted profile (21 variables) and full profile (43 variables). In addition, in an innovative initiative, this edition integrates demonstrative instructional videos accessible via QR codes [7]. Currently, ISAK comprises an international network with over 36,000 accredited anthropometrists in 89 countries. Recently, ISAK introduced the *ISAK-Metry* software as a tool to assess body composition and derived parameters based on anthropometric properties.

Collectively, this historical record clearly indicates that anthropometric standardization developed primarily through pluralistic and complementary international academic initiatives rather than through a single centralized institutional process. Despite this, such an interpretation is not unanimously supported in recent literature. Silva and Vieira [56], in a previous report on the ISAK accreditation scheme, described the ASRM [6] as an integrative and systematized proposal derived from anthropometric measurement procedures previously established by IWGK in 1985. However, the available historical evidence does not support this interpretation. Although the 1980s were marked by substantial theoretical debate on anthropometric standardization, the evidence suggests that ASRM [6] was conceived and developed independently, without formal institutional or methodological ties to the IWGK. Furthermore, there is no documented evidence to support the notion that disagreements between the groups precipitated the IWGK-ISAK transition in 1986 [[6], [7], [8],^10^].

Having outlined the historical development and institutional standardization of skinfold protocols and standards, the following subsection presents a descriptive synthesis of the literature examining how these technical procedures have been operationalized in research and practice.

### Skinfold measurement: technical and methodological synthesis of protocols and standards

A targeted bibliographic synthesis was conducted using classic textbooks, consensus manuals, and seminal peer-reviewed studies on surface anthropometry. Matiegka’s [25] work was treated as an early landmark for body-compartment conceptualization. Across the included sources, 17 skinfold sites were identified (2 on the head, 8 on the trunk, 3 on the upper limbs, and 4 on the lower limbs), along with 31 distinct technical descriptions (22 site-selection protocols and 9 measurement standards). Tables 1 and 2 summarize the frequency of skinfold sites reported across historical and contemporary anthropometric protocols and standards, respectively, highlighting the variability and recurrence of anatomical site selection over time. Although several sites and reference landmarks are traditionally designated using anglicized terms (e.g., subscapular and supraspinal), the standard proposed by Esparza-Ros et al. [7], within the ISAK framework, adopts some terms aligned with the Latin-based “*Terminologia Anatomica*” (e.g., subscapulare and supraspinale). However, given the terminological divergences between protocols and standards, all skinfold sites designated throughout this review have been standardized in English to preserve conceptual clarity and comparative interpretation between different methodological frameworks.

**TABLE 1:** Frequency of skinfold sites described in anthropometric protocols

References	Skinfold sites																	Total
	Head		Trunk								Limbs							
	Cheek	Chin	Chest	Subscapular	Thorax	Axillary	Waist	Abdominal	Iliac crest	Supraspinal	Triceps	Biceps	Forearm	Thigh	Buttock	Knee	Calf	
Matiegka [25]					✓			✓				✓	✓	✓			✓	6
Brozek and Keys [29]			✓	✓				✓			✓					✓		5
Keys and Brozek [27]				●					✓		●							1
Skerlj et al. [30]		✓	✓	✓		✓	✓	✓			✓			✓		✓	✓	10
Edwards et al. [31]				●	✓						●	●						1
Hammond [32]				✓	✓			✓	✓		✓	✓						6
Keys [33]			✓	✓							✓							3
Pascale et al. [34]			✓	✓		✓	✓	✓	✓		✓							7
Allen et al. [35]	✓	●	●	●		●	●	●			●			●		●	●	1
Young et al. [37]		✓	✓	✓	✓	✓	✓	✓	✓		✓			✓		✓		11
Sloan et al. [39]								✓	✓		✓			✓				5
Durnin and Rahaman [40]				✓					✓		●	✓						3
Sloan [41]			✓	✓				✓	✓		✓			✓	✓			7
Heath and Carter [42]				✓						✓	✓						✓	4
Katch and Michael [43]			✓	✓				✓	✓		✓							5
Michael and Katch [44]			✓	✓				✓	✓		✓			✓				6
Wilmore and Behnke [45]				✓		✓		✓	✓					✓		✓		6
Parizková and Roth [46]	✓	✓		✓	✓	✓		✓	✓		✓	✓				✓	✓	11
Johnston et al. [47]				✓		✓			✓		✓						✓	5
Lohman [49]			✓	✓		✓	✓	✓			✓			✓		✓	✓	10
Sinning et al. [50]			✓	✓		✓		✓	✓	✓	✓			✓				8
Jackson and Pollock [51]			✓	✓		✓		✓	✓		✓			✓				7
Total	2	3	11	17	5	9	4	15	14	2	16	4	1	10	1	6	6	

Note: ✓ (Site described); ● (Site cited).

**TABLE 2:** Frequency of skinfold sites described in anthropometric standards

References	Skinfold sites																	Total
	Head		Trunk								Limbs							
	Cheek	Chin	Chest	Subscapular	Thorax	Axillary	Waist	Abdominal	Iliac crest	Supraspinal	Triceps	Biceps	Forearm	Thigh	Buttock	Knee	Calf	
ICNND [36]			✓	✓	✓			✓			✓							5
Tanner and Whitehouse [38]				✓							✓							2
Weiner and Lourie [3]			✓	✓		✓		✓	✓		✓	✓	✓	✓			✓	10
Behnke and Wilmore [4]		✓	✓	✓		✓	✓	✓	✓		✓			✓		✓	✓	11
Parizkova [48]	✓	✓	✓	✓		✓		✓	✓		✓					✓	✓	10
Ross and Marfell-Jones [5]				✓				✓	✓	✓	✓	✓		✓			✓	8
Lohman et al. [6]			✓	✓		✓		✓	✓		✓	✓	✓	✓		✓	✓	11
Norton and Olds [58]				✓		✓		✓	✓	✓	✓	✓		✓			✓	9
Esparza-Ros et al. [7]				✓				✓	✓	✓	✓	✓		✓			✓	8
Total	1	2	5	9	1	5	1	8	7	3	9	5	2	6	0	3	7	

Note: ✓ (Site described).

Abbreviation: ICNND, Interdepartmental Committee on Nutrition for National Defense.

Subscapular (83.9%), triceps (80.6%), abdominal (74.2%), and iliac crest (67.7%) were the most frequently reported anatomical sites used in body composition assessment protocols [38,40,45,49], reference manuals [[3], [4], [5], [6]], and international anthropometric standardization systems [7,[59], [60], [61]]. Classic cadaver-based analyses corroborate this predominance, supporting these regions as major subcutaneous fat depots [15]. The chest (51.6%), thigh (51.6%), axillary (45.2%), and calf (41.9%) sites, although relevant, show less consensus in reports. This variability is partially explained by greater tissue compressibility [15] and by differences in the relative proportions of superficial and deep subcutaneous adipose tissue [16], which may reduce interrater reproducibility, particularly in the mid-thigh [15,16]. The thorax site, despite its anatomical proximity to the chest, shows substantially lower frequency and is not consistently incorporated into most protocols. Lastly, sites such as the biceps, knee, supraspinal, cheek, and forearm appear in <30% of studies and are typically used in exploratory studies or context-specific applications [28,41,42,49]. The buttocks region, described by Sloan [41], was rarely adopted in later protocols, likely because of limited acceptability and practical constraints related to privacy and participant comfort.

Reporting frequency was assessed as the number of times each skinfold site was reported across the included protocols and standards. Thus, the results indicate that the triceps and subscapular skinfolds are among the most frequently described and methodologically consolidated sites in the specialized literature and constitute robust anthropometric indicators with simplified application for epidemiological surveillance and longitudinal monitoring of regional adiposity patterns. Their restricted use may be particularly useful in clinical contexts with operational limitations, where the assessment of body composition through multiple skinfold measurements is not feasible and can be complemented using *z-scores* as an appropriate tool for standardization and individual follow-up [8]. In this context, anatomical definitions of skinfold sites show substantial variability across references. More comprehensive standards, such as those by Behnke and Wilmore [4] and Lohman et al. [6], report a larger number of skinfold sites, whereas older works and studies with more specific objectives describe only 1 or a few sites, as in Keys and Brozek [27] and Allen et al. [35].

**FIGURE 2:**
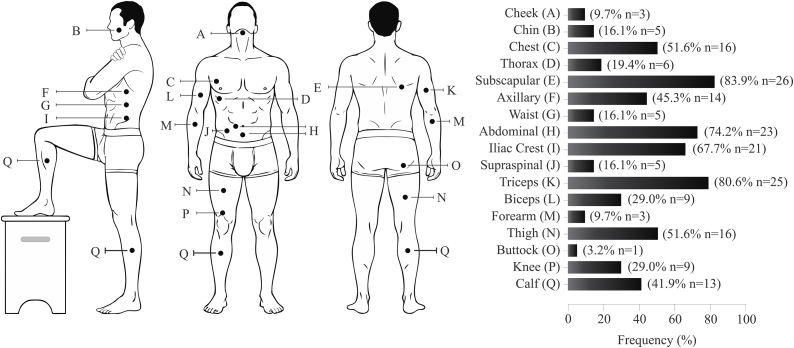
Frequency of skinfold sites described in the specialized literature.

Figure 2 illustrates this morphological heterogeneity in the distribution of skinfold sites and the methodological diversity present in the literature, which is consistent with the marked differences in site prevalence observed across studies. Furthermore, most sites are associated with >1 distinct anatomical description [6], such as the abdominal, iliac crest, and chest sites. However, in the present review, only the standardized skinfold measurement procedures proposed by Weiner and Lourie [3], Behnke and Wilmore [4], Ross and Marfell-Jones [5], Lohman et al. [6], and, finally, Esparza-Ros et al. [7] are discussed (Table 3). These approaches represent the most consolidated standards, with wide acceptance and continued relevance in scientific research and clinical practice contexts. Taken together, the 5-skinfold standardization systems analyzed show a high degree of convergence in the selection of anatomical sites. This alignment reflects a historical consensus concerning the body regions that best represent subcutaneous adiposity, as well as the predominantly vertical orientation of skinfolds on the limbs, based on the longitudinal alignment of body segments and the use of easily identifiable bony landmarks.

**TABLE 3:** Description of the anatomical location of skinfold sites according to the 5 main anthropometric standards

Reference						
		Weiner and Lourie [3]	Behnke and Wilmore [4]	Ross and Marfell-Jones [5]	Lohman et al. [6]	Esparza-Ros et al. [7] 1
Head	Chin		Vertical fold below the mandible between the chin and neck.			
Trunk	Chest	Oblique fold laterally and at the same level as the nipple.	Oblique fold over the lateral border of the pectoralis major muscle, just medial to the axilla.		Oblique fold 1 cm below the highest point of the anterior axillary crease.	
	Subscapular	Vertical fold below the angle of the scapula.	Oblique fold at the inferior angle of the scapula, parallel to the axillary border.	Diagonal fold at the inferior angle of the scapula.	Diagonal fold just below the inferior angle of the scapula, inclined inferolaterally ∼45° to the horizontal plane in the natural cleavage lines of the skin.	Diagonal fold 2 cm along a line running laterally and obliquely downward from the *scapulare* landmark at a 45° angle.
	Axillary	Vertical fold on the midaxillary line at the level of the xiphoid process.	Vertical fold in the midaxillary line at the level of the 5th rib.		Horizontal fold in the midaxillary line at the level of the xiphisternal junction.	
	Waist		A vertical fold in the midaxillary line, midway between the 12th rib and the iliac crest.			
	Abdominal	Vertical fold 2 cm to the left of the level of the umbilicus.	Horizontal fold adjacent to the umbilicus.	Vertical fold 5 cm laterally and at the level of the midpoint of the umbilicus.	Horizontal fold 3 cm laterally and 1 cm inferiorly of the midpoint of the umbilicus.	Vertical fold 5 cm horizontally to the right-hand side of the *omphaliom*.
	Iliac crest	Vertical fold 1 cm above and 2 cm medially to the anterior superior iliac spine.	Vertical fold on the iliac crest, in the midaxillary line.	Horizontal fold immediately above the iliac crest in the midaxillary line.	Oblique fold immediately superior to the iliac crest in the midaxillary line.	Horizontal fold immediately above the marked *iliocristale*.
	Supraspinal			Diagonal fold at the intersection of the ilium border on a line from the spine to the anterior axillary border.		Diagonal fold at the intersection of the line from iliospinale to the anterior axillary border, and the horizontal line from the *ilocristale*.
Limbs	Triceps	Vertical fold on the posterior aspect of the arm, 1 cm above the landmark for the arm circumference and aligned directly with the olecranon.	Vertical fold midway between the acromial and olecranon processes on the posterior aspect of the arm.	Vertical fold on the *mid-acromiale-radiale* line on the posterior aspect of the arm.	Vertical fold in the midline of the posterior aspect of the arm, at a point midway between the lateral projection of the acromion process of the scapula and the inferior margin of the olecranon process of the ulna.	Vertical fold on the posterior surface of the arm, in the midline at the level of the marked *mid-acromiale-radiale*.
	Biceps	Vertical fold on the anterior aspect of the arm, directly above the center of the cubital fossa.		Vertical fold on the *mid-acromiale-radiale* line on the anterior aspect of the arm.	Vertical fold 1 cm above the line marked for the triceps skinfold and the circumference of the arm on a vertical line joining the anterior border of the acromion and the center of the antecubital fossa.	Vertical fold on the anterior surface of the arm at the level of the mark projected forward from the *mid-acromiale-radiale*, on the midline of the biceps muscle belly.
	Forearm	Vertical fold on the lateral aspect of the forearm and at the midpoint of the radius.			Vertical fold on the posterior aspect of the forearm at the level of the maximum circumference.	
	Thigh	Vertical fold on the anterior aspect of the thigh, midway between the midpoint of the inguinal crease and the superior border of the patella.	Vertical fold on the anterior aspect of the thigh midway between the hip and knee joints.	Vertical fold on the anterior aspect of the thigh, midway between the inguinal crease and the anterior patella.	Vertical fold on the anterior aspect of the thigh, midway between the midpoint of the inguinal crease and the proximal border of the patella.	Vertical fold at the midpoint of a line between the *patellare* and the inguinal point.
	Knee		Vertical fold at the midpoint of the patella.		Vertical fold on the anterior aspect of the thigh, 2 cm proximal to the proximal edge of the patella.	
	Calf	Vertical fold on the medial aspect of the calf at the level of maximum circumference.	Vertical fold on the posterior aspect of the calf at the level of the maximum circumference.	Vertical fold on the medial aspect of the calf at the level of maximum circumference.	Vertical fold on the medial aspect of the calf at the level of maximum circumference.	Vertical fold on the most medial aspect of the calf at the level of the maximal girth.

The anthropometric terminology used in each reference has been kept as originally reported to ensure a faithful representation of the authors’ methodological approaches.

1 Anthropometric standard proposed by the *International Society for the Advancement of Kinanthropometry*.

Because measurement sites on the trunk, particularly the subscapular, chest, and iliac crest, often differ in reference points and fold orientation across anthropometric standards, the same site designation can generate systematically different values, thus limiting comparability between studies and the interpretative coherence of cross-sectional or longitudinal analyses. A concrete representation of the methodological and practical consequences of such anatomical inconsistency is observed in the interchangeable use of the supraspinal and iliac crest skinfolds within the Heath–Carter somatotype method. Comparative investigations have documented systematic differences between these 2 anatomical sites, with the iliac crest consistently producing greater thickness values. When replaced by the supraspinal site, this discrepancy results in an overestimation of endomorphism that can distort somatotype classification [62]. Although regression-based correction models can partially mitigate distortions arising from incorrect site identification, strict adherence to anatomically defined standards remains methodologically indispensable to ensure that the measured values accurately reflect the intended body component and maintain analytical consistency.

Such interstandard discrepancies reflect distinct morphological, structural, and functional interpretations related to skin and subcutaneous adipose tissue. Variation in topographic precision is observed, as some classic approaches, such as those proposed by Weiner and Lourie [3] and Behnke and Wilmore [4], present general anthropometric descriptions, whereas others provide detailed geometric definitions with standardized anatomical distances and/or specified angles, as proposed mainly by Lohman et al. [6] and Esparza-Ros et al. [7]. Thus, the documented technical and methodological aspects related to each skinfold site are available in Supplementary Material.

In this review, skinfold measurement sites show considerable heterogeneity regarding frequency of use, anatomical definition, and fold orientation, with greater consensus observed in widely adopted sites and persistent ambiguity in less frequently used sites. Craniofacial sites are poorly documented: the cheek skinfold is rarely included, and existing descriptions converge on a horizontal fold at the level of the nostrils, whereas the chin skinfold is more consistently defined as a vertical fold below the mandible, reflecting the vertical orientation of the local musculature. Trunk skinfolds sites predominate in protocols and standards, particularly the subscapular, axillary, thorax, abdominal, and iliac crest, although discrepancies persist regarding precise reference points and fold direction.

Despite this variability, patterns generally converge toward aligning skinfold orientation with the direction of underlying muscle fibers, favoring oblique folds in regions dominated by obliquely oriented muscles (e.g., pectoralis major, subscapular region, and external oblique) because of better fold formation and less discomfort for the participant. Limb skinfolds, especially triceps, thigh, and calf, show the highest level of standardization, with consistent landmarks and vertical folds aligned with longitudinal muscle fibers. In contrast, less frequently used sites, such as the chest, forearm, buttocks, and knee, have limited documentation and weaker consensus, often reflecting practical limitations, invasive nature, or questionable benefit for assessing total body adiposity. Overall, the evidence highlights a clear gradient of methodological consolidation, where commonly used skinfold measurements are characterized by strong agreement between standards, while rarely applied sites remain descriptively incomplete or methodologically inconsistent, underscoring the need for clearer standardization or justification for their inclusion in contemporary anthropometric practice.

Although most anthropometric standards do not explicitly refer to skin tension lines (*Langer’s lines*), the orientation of several trunk skin folds appears to coincide not only with the direction of the underlying muscle fibers but also with the skin’s natural dermal tension lines. Such alignment may facilitate fold formation, reduce mechanical resistance during pinching, and potentially increase the comfort and consistency of the procedure. Although these anatomical considerations are not formally incorporated into classic standardization manuals, they may partially explain the historical convergence toward oblique or vertical orientations in specific anatomical regions [7,12].

The anthropometric standards discussed in this narrative review converge on the general technical principles of skinfold thickness measurement: *1*) perform the procedures on the right side of the individual’s body; *2*) use an appropriate skinfold caliper under favorable calibration conditions; *3*) pinch the fold between the tips of the left thumb and index finger; *4*) hold the caliper with the right hand and apply the jaws perpendicularly to the fold; *5*) control the reading time; and *6*) keep the fold elevated until the measurement is complete [[3], [4], [5], [6], [7]]. The observed differences reflect the methodological evolution of applied anthropometry over the last few decades. Weiner and Lourie [3] and Behnke and Wilmore [4] focused on basic operational description. Notably, these early standards were adopted methodologically in the development of classic skinfold-based regression equations, including those proposed by Durnin and Womersley [52] and Jackson and Pollock [53], respectively, which remain widely applied in clinical and sports contexts [63].

In turn, Ross and Marfell-Jones [5] introduced greater technical rigor to measurement procedures, such as the recommendation to use specific calipers and pinch the skinfold at the designated location, applying the caliper jaws 1 cm from the fingers. These refinement processes culminated in more comprehensive and systematized standardization proposals, paradigmatically represented by Lohman et al. [6] and Esparza-Ros et al. [7]. Thus, our analysis is limited to these 2 bibliographic references, as they synthesize the historical conceptual foundations and contemporary technical standardization of skinfold measurement.

A further relevant distinction concerns the updating dynamics and accessibility of these reference documents. The standard proposed by Lohman et al. [6] was originally published in 1988 and revised in 1991 [64], without subsequent substantial updates. Furthermore, most classic anthropometry textbooks [3,4,5,57,58] that underpin contemporary skinfold technique methodology were also published in print during the 20th century, and access to them is mainly limited to specialized university libraries or rare commercial copies in second-hand bookstores. This historical pattern reflects a normative consolidation model characterized by long-standing conceptual stability but also imposes structural limitations on methodological transparency and reproducibility in different research and field contexts. In contrast, the standard proposed by Esparza-Ros et al. [7], represented by ISAK, follows an iterative model of procedural refinement, with periodic revisions that incorporate contemporary quality control requirements. However, despite its ongoing updating process, access to the digital technical manual is restricted to active ISAK members through formal accreditation. Thus, classic and contemporary anthropometric standards present distinct, yet relevant, barriers to universal accessibility, whether due to limited file availability or institutional access control, which can indirectly restrict standardization efforts and comparability between studies.

Recent evidence has highlighted substantial heterogeneity in the development of skinfold-based regression equations [65], which reinforces the need to base the present discussion on consolidated and systematically established standardization frameworks. By adopting these references as our analytical axis, we align with internationally recognized protocols, thereby enhancing the comparability, reproducibility, and external validity of skinfold-based body composition assessments. An important additional structural difference between these contemporary standards concerns the number and distribution of skinfold sites included in their respective protocols. Lohman et al. [6] describe 11 skinfold sites, whereas Esparza-Ros et al. [7] define 8 sites within their normative framework.

Notably, Lohman et al. [6] propose a broader anatomical coverage based on specific segmental sites, such as the knee and forearm, and thoracic sites, such as the chest and midaxillary. However, although the chest skinfold has been incorporated into classic regression equations, such as those proposed by Jackson and Pollock [53] and Jackson et al. [54], its inclusion has not been consistently maintained in recent studies [13]. Furthermore, the chest skinfold presents greater operational complexity, particularly in female populations, because of the increased invasiveness associated with using the nipple as an anatomical reference during the procedures for identifying and marking the measurement site, which may affect participant comfort and assessment feasibility. In contrast, Esparza-Ros et al. [7] propose a balanced topographic model comprising 4 trunk sites (subscapular, iliac crest, supraspinal, and abdominal) and 4 limb sites (triceps, biceps, thigh, and calf), thereby ensuring proportional representation of central and appendicular subcutaneous adiposity. This configuration is supported by the recent development of population reference percentile curves (third, 10th, 25th, 50th, 75th, 90th, and 97th) for the sum of these 8 skinfolds across age- and sex-specific groups [14].

The anthropometric standardization proposed by Lohman et al. [6] has been consolidated since the 20th century as the main technical-methodological reference for applied kinanthropometry, systematizing the anatomical, statistical, and population fundamentals of skinfold measurements, with wide influence on research protocols and clinical guidelines, such as those proposed by the *American College of Sports Medicine*. Furthermore, although the *NHANES* does not explicitly cite a specific anthropometric standard adopted in its guidelines [66], the technical procedures for measuring triceps and subscapular skinfolds are consistent with those described by Lohman et al. [6]. Notably, these authors present an important discussion on the formation, orientation, and manipulation of skinfolds. However, although marking anatomical reference sites is considered optional in many contexts, emphasizing its formative and exploratory character [6], it is well documented in the literature that skinfold measurement is preceded by marking their respective sites [12,18].

The anthropometric standardization proposed by Esparza-Ros et al. [7] represents a contemporary, normative, and operational approach, specifically oriented toward controlling technical measurement error, with emphasis on verifying instrumental calibration, systematic landmarking of anatomical sites, standardization of reading time, and serial repetition of measurements. Thus, although the classical standard prioritizes general principles and allows greater technical flexibility depending on the anthropometrist’s experience, the contemporary standard shifts the focus to reproducibility, interrater comparability, and quality control, responding to current demands for international standardization and methodological robustness.

**FIGURE 3:**
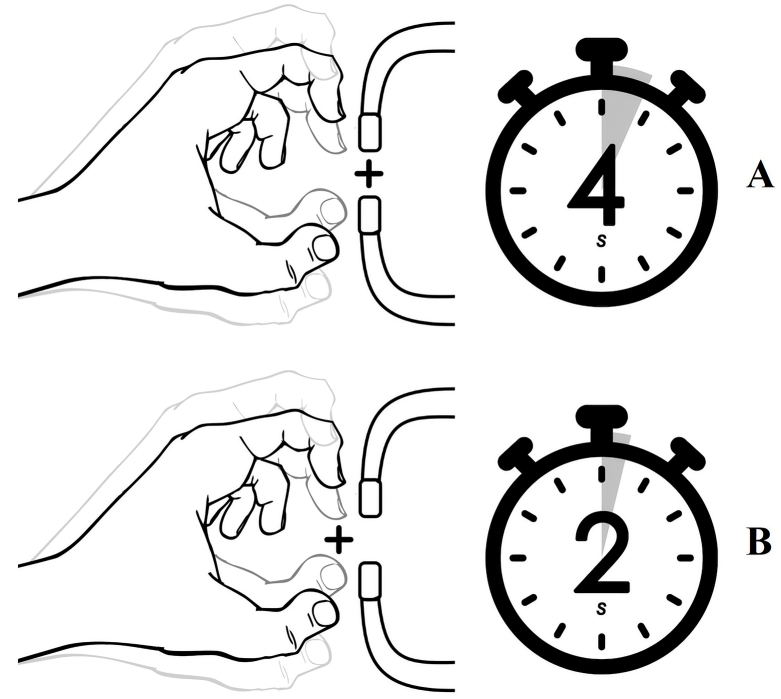
Spatial and temporal differences between 2 standardized skinfold measurement approaches. (A) Lohman et al. [6]: the skinfold is pinched ∼1 cm proximal to the marked anatomical site, the caliper jaws are applied directly over the site, and the measurement is read 4 s after full caliper pressure is applied. (B) Esparza-Ros et al. [7]: the skinfold is pinched directly at the marked anatomical site, the caliper jaws are positioned ∼1 cm distal to the site, and the measurement is read 2 s after full caliper pressure is applied.

The main technical differences between these skinfold measurement standards [6,7] lie in 2 dimensions: the *spatial dimension* (skinfold pinching and caliper application) and the *temporal dimension* (reading the measurement). As illustrated in Figure 3, Lohman et al. [6] recommend pinching the skinfold 1 cm proximal to the previously defined anatomical site, positioning the caliper jaws directly over this site, and reading the measurement 4 s after applying full caliper pressure (Figure 3A). In contrast, Esparza-Ros et al. [7] recommend pinching the skinfold directly at the previously defined anatomical site, positioning the caliper jaws 1 cm distal to this site, and reading the measurement 2 s after applying full caliper pressure (Figure 3B).

Hume and Marfell-Jones [18] indicated that skinfold measurements exhibit high spatial variability, and most sites are sensitive to small anatomical variations. From a technical-methodological perspective, it is assumed that the designated site represents, in theoretical and population terms, the point at which subcutaneous adipose tissue is morphologically most pronounced. Consequently, measuring skinfold thickness directly at the defined anatomical site (i.e., precisely at the intersection between the marked reference lines), as proposed by Lohman et al. [6], seems to be the most appropriate practical approach, as it reinforces the conceptual coherence between site definition, skinfold pinching, and caliper jaw application. Regarding temporal control, the recommendation to read after 2 s (i.e., at the third second), proposed by Esparza-Ros et al. [7], is supported by subsequent empirical evidence. Vaquero-Cristóbal et al. [19] found time-dependent changes in skinfold thickness, with stabilization generally occurring between 1.5 and 2.5 s depending on the site and sex, and the minimum values frequently observed between ∼2.2 and 3 s.

In practical terms, although the 4-s reading recommended by Lohman et al. [6] may provide additional assurance that tissue compression has fully stabilized, available empirical evidence indicates that most anatomical sites reach near-minimal thickness between ∼2 and 3 s after full caliper pressure is applied [19]. Extending compression beyond this interval may increase viscoelastic deformation of the subcutaneous adipose tissue, potentially resulting in slightly lower values due to continued tissue displacement. Conversely, reading performed too early may capture measurements during the transient compression phase. Therefore, from a methodological standpoint, a reading obtained ∼2 to 3 s after full caliper pressure appears to represent a balanced and evidence-informed approach, reconciling physiological stabilization with controlled compression duration. Regardless of the selected interval, strict temporal standardization is essential to ensure measurement comparability and test–retest reliability. Machado et al. [67] have shown that the evaluator’s experience influences the reliability of skinfold measurements and the estimation of body fat percentage. Whereas the experienced evaluator presented technical measurement errors within acceptable limits (<5%), the inexperienced evaluator exceeded these limits in more complex skinfolds, such as iliac and abdominal folds, resulting in low agreement in the estimation of body fat. The results reinforce the need for rigorous training and continuous quality control to ensure accurate anthropometric assessments.

Operational factors related to site standardization can influence skinfold measurement, as observed for the subscapular, abdominal, and mid-thigh skinfolds. For the subscapular skinfold, Lohman et al. [6] and Esparza-Ros et al. [7] describe a diagonal fold oriented ∼45° inferolaterally. However, Lohman et al. [6] position the site immediately below the inferior angle of the scapula, whereas Esparza-Ros et al. [7] specify a displacement of 2 cm along an oblique line from the scapular landmark. Although seemingly minor, this difference in landmarking may affect local tissue composition, as small positional shifts can alter the relative contribution of skin, subcutaneous fat, and underlying musculature, potentially influencing skinfold compressibility and thickness [12,15,16,18].

For the abdominal site, more pronounced differences are evident. Lohman et al. [6] recommend a horizontal fold located 3 cm laterally and 1 cm inferior to the umbilicus, whereas Esparza-Ros et al. [7] propose a vertical fold positioned 5 cm laterally to the umbilical landmark. These variations involve both orientation and site location, which are critical factors in skinfold assessment. Fold orientation relative to natural skin cleavage lines and underlying fiber direction can influence fold formation and tissue compression. In the abdominal region, the predominantly transverse orientation of skin tension lines and the longitudinal orientation of the rectus abdominis fibers may favor the elevation and stabilization of a vertical fold during pinching. Additionally, increasing the lateral distance from 3 to 5 cm may shift the measurement to regions with different fat distribution patterns, particularly in individuals with higher central adiposity [12,15,16,18]. In pediatric populations, however, Esparza-Ros et al. [7] propose a proportional adjustment of this lateral distance based on stature (cm), calculated as $\frac{\left(5\times stature\right)}{170.18}$, to account for body size differences. As a result, these methodological discrepancies can introduce systematic variation and limit the comparability of abdominal skinfold measurements between different standardizations.

Body position also represents a relevant operational factor, particularly for the mid-thigh skinfold. Lohman et al. [6] recommend that the measurement be performed with the individual standing. Body weight is transferred to the left foot while the right leg is relaxed, with the knee slightly flexed and the foot flat on the floor. In contrast, Esparza-Ros et al. [7] recommend that the individual be seated with the knee flexed at ∼90°. These distinct recommendations likely reflect different biomechanical considerations related to tissue stabilization and ease of skinfold formation. In the standing position, the quadriceps muscles remain under postural tension, which can increase skin tension and hinder the formation of a stable skinfold, especially in individuals with greater muscle development. On the other hand, knee flexion in the seated position tends to reduce quadriceps tension and promote greater relaxation of the skin–subcutaneous adipose tissue complex, facilitating the gripping and stabilization of the skinfold during caliper application. Consequently, measurements obtained under different postural conditions may not be strictly interchangeable, because variations in muscle tension and tissue compressibility can introduce systematic differences in skinfold thickness values.

Finally, Lohman et al. [6] and Esparza-Ros et al. [7] converge on the recommendation that all skinfold measurements be systematically performed on the right side of the body. This convention aimed to reduce interindividual variability related to potential bilateral asymmetries and to ensure consistency across assessments, thereby improving comparability among evaluators, protocols, and studies. Empirical evidence indicates no significant bilateral differences in subcutaneous adiposity, suggesting that side selection has minimal impact on body composition estimates when standardized procedures are followed [68]. However, in sporting contexts characterized by unilateral predominance, where muscle hypertrophy may differ between sides, or in disciplines where limb symmetry is particularly relevant, such as bodybuilding, it is recommended that girth measurements be taken on both sides to provide a more accurate and context-sensitive assessment [6,7,68].

Future directions in skinfold thickness assessment should prioritize harmonizing existing standards through the development of internationally accepted protocols that clearly define anatomical landmarks, skinfold orientation, and measurement procedures. This effort could be supported by consensus initiatives and collaborative working groups aimed at reducing variability between studies and improving comparability across different contexts. In parallel, advancements in skinfold caliper technology should focus on improving mechanical consistency, including standardized calibration procedures, controlled jaw pressure according to the type of physical-mechanical configuration, and transparent disclosure of instrument specifications. Further research is also needed to better characterize tissue compressibility, considering factors such as hydration status, age, sex, and regional anatomical variation, which can influence measurement accuracy.

Another key priority is the development of predictive models based on population-specific data from large and diverse datasets, incorporating contemporary statistical approaches and external validation. These models must be rigorously tested across different ethnicities, age ranges, and body composition profiles to enhance their generalizability and clinical applicability. Finally, strengthening training systems and quality control procedures is essential. This includes implementing standardized certification programs, conducting regular technical error of measurement (TEM) assessments, monitoring interobserver and intraobserver reliability, and using digital tools to support training and auditing processes.

Importantly, skinfold measurements should be reframed not only as indirect predictors of total body fat but also as markers of regional subcutaneous adiposity distribution. Together, these efforts aim to enhance methodological rigor, technological precision, interpretative clarity, and clinical relevance, in addition to maintaining the practicality and accessibility that make the technique so widely used. Further studies are needed to evaluate training programs by comparing different protocols and their effects on interobserver error. Such efforts may help establish site-specific TEM values for each skinfold site.

In conclusion, skinfold measurement protocols and standards are not interchangeable and reflect pluralistic, complementary international initiatives rather than a single institutional standardization process. Although there is substantial convergence on general technical principles, important divergences persist, particularly in the definition and anatomical location of trunk skinfold measurement sites. Each approach has its own methodological assumptions, as well as advantages and limitations. Subtle differences in procedures can introduce systematic variability and compromise comparability between studies, especially when technical criteria are not explicitly described and reported.

Collectively, the evidence indicates that the validity of body composition estimates derived from anthropometric properties depends on strict adherence to standardized and clearly defined general measurement procedures. Given that the main skinfold-based regression equations were predominantly developed using a Type A or Type B skinfold caliper, the instrument available in the field will determine which predictive model will be employed. Once the regression equation corresponding to the skinfold caliper to be used is selected, the skinfold thickness should be measured rigorously according to the technical procedures described by the study authors. In this sense, the interpretation of the results should preferably be based on normative reference values developed with the same measurement standard used. Furthermore, adequate training of anthropometrists under expert supervision, combined with rigorous quality control procedures, is essential to ensure measurement reliability. However, the interpretative limitations inherent to the skinfold method must also be acknowledged.

On the basis of the available literature, it is not methodologically justifiable to define a universally superior skinfold measurement protocol or standard. Its suitability, therefore, depends on the context and the consistency with which it is applied. Nonetheless, within this contextual perspective, the contemporary standard proposed by ISAK may represent a pragmatic reference framework, as it reflects an internationally consolidated consensus sustained by continuous revision and structured accreditation. Finally, in a field historically marked by procedural heterogeneity, such institutional cohesion may facilitate global standardization and enhance methodological transparency in skinfold-based assessments.

## Author contributions

The authors’ responsibilities were as follows – JHC: designed the research and wrote the manuscript; JHC, JPC-P, FAJ: conducted the research; WLR, MIF-C, FAJ: analyzed data; AMS, LBS, TGL, SBH: critically reviewed and edited the manuscript; and all authors: read and approved the final manuscript.

## Declaration of Generative AI and AI-Assisted Technologies in the Writing Process

The authors declare that no generative AI and AI-assisted technologies were used in the writing of this manuscript.

## Funding

JHC is partially funded by the *Fundação Cearense de Apoio ao Desenvolvimento Científico e Tecnológico**,* Brazil. JPC-P is partially funded by the *European Society for Clinical Nutrition and Metabolism* *Broaden Your Horizons* *Grant 2025* and *Coordenação de Aperfeiçoamento de Pessoal de Nível Superior*, Brazil. The supporting sources are not involved or have any restrictions on this publication.

## Conflict of interest

JHC is a Level 3 instructor at the International Society for the Advancement of Kinanthropometry. JPC-P has received speaking fees/honoraria from Fresenius Kabi, Danone Nutricia Brazil, and Prodiet Medical Nutrition. SBH serves on the Medical Advisory Boards of Tanita Corporation, Novo Nordisk, Abbott, Novartis, Versanis, and Medifast.
