# Supplementary material for: Skinfolds Measurement Protocols and Standards: A Narrative Review
Source: Adv Nutr. 2026 May 26;17(7):100662. doi: 10.1016/j.advnut.2026.100662 (PMC13276579; doi:10.1016/j.advnut.2026.100662)
Supplement: Multimedia component 1 [file mmc1.docx]

**Supplementary Material**

**Review**

**Skinfolds measurement protocols and standards: a narrative review**

Joaquim H Cintra*, Jarson P Costa-Pereira, Wagner L Ripka, Maria I Freire-Correia, Filipe A Jesus, Analiza M Silva, Luís B Sardinha, Timothy G Lohman and Steven B Heymsfield

* Corresponding author.

E-mail address: [joaquimcintra@hotmail.com](mailto:joaquimcintra@hotmail.com) (J.H. Cintra)

This supplementary material provides a structured synthesis of the characteristics of the skinfold sites identified in this review. Each site is described based on the frequency of inclusion in protocols and standards, anatomical definition, fold orientation, key technical considerations, and methodological considerations. Anatomical sites are presented individually (A–Q) to facilitate comparisons.

A) *Cheek skinfold site*: only three documents included this skinfold site, two protocols [35,46] and one standard [48]. Standards converge in indicating a *horizontal* fold in the cheek region, at the level of the nostrils, but differ in methodological precision. Allen et al. [35] describe the location below the right temple. Parizkova and Roth [46] and Parizkova [48] are more specific in defining it at the midpoint of the line connecting the tragus to the nostrils.

B) *Chin skinfold site*: this skinfold site is considered by five documents, three protocols [30,37,46], two standards [4,48], and cited by one protocol [35]. According to the standard by Benke and Wilmore [4], this site is described as the *vertical* fold below the mandible, between the chin and the neck, with no differences compared to other documents [30,46]. As most muscles in this site are vertically orientated, the alignment of this skinfold is vertical accordingly.

C) *Chest skinfold site*: this skinfold site is presented in 16 documents, namely 11 protocols [29,30,33,34,37,41,43,44,49-51], five standards [3,4,6,33,48], and cited by one protocol [35]. The standards differ regarding the precise location of this site: Weiner and Lourie^3^ placed the fold at the nipple level, whereas Lohman, Roche, and Martorell defined it as 1 cm below the highest point of the anterior axillary crease [6]. However, the standards [3,4,6] agree on the direction and general location of the skinfold, describing it as an oblique fold along the lateral border of the pectoralis major muscle and medial to the axillary crease. This orientation follows the oblique alignment of the pectoralis major muscle fibers, which guides the direction of the fold.

D) *Subscapular skinfold site:* this skinfold site was the most frequent across the included studies, accounting for 25 documents, of which 17 were protocols [29,30,32–34,37,40–47,49–51], eight standards [3–7,38,48,58], and cited by three protocols [27,28,35]. Overall, most standards agree on locating the skinfold site below the inferior angle of the scapula [3–6]. In contrast, Esparza-Ros, Vaquero-Cristóbal and Marfell-Jones [7] specify a more detailed positioning, indicating that the site should be located 2 cm along a line extending laterally and obliquely downward from the inferior angle of the scapula. Regarding fold orientation, Weiner and Lourie [3] and Lohman, Roche and Martorell [6] describe a vertical fold, whereas Behnke and Wilmore [4], Ross and Marfell-Jones [5], and Esparza-Ros, Vaquero-Cristóbal and Marfell-Jones [7] recommend an oblique fold. Considering the oblique orientation of muscle fibers in this region, an oblique fold may be more easily grasped and associated with less discomfort for the assessed individual, particularly when taken at a short distance from the inferior angle of the scapula.

E) *Thorax skinfold site*: this skinfold site was included in six documents, five protocols [25,28,32,37,46] and one standard [36]. There is considerable variation in the anatomical definition of this site. One protocol [32] places it immediately below the costal margin in line with the nipple, whereas another [25] locates it midway between the nipples and the umbilicus at the level of the costal margin. The ICNND standard [36] describes only a site adjacent to the nipple, without precise landmarks. In contrast, Parizkova and Roth [46] define a slightly oblique horizontal fold above the 10^th^ rib at the anterior axillary line. Given the orientation of the ribs and intercostal muscles, a fold aligned with the ribs may be more anatomically consistent and reproducible than a strictly vertical fold in this region.

F) *Axillary skinfold site*: this skinfold was described in 12 documents, including nine protocols [30,34,37,45–47,49–51] and three standards [3,4,48]. It was also cited in one additional protocol [35]. Although the standards consistently locate the site along the midaxillary line, they differ in its precise position: at the level of the xiphoid process [3], the fifth rib [4], or the xiphosternal junction [6]. They also differ in fold orientation, with Weiner and Lourie [3], as well as Behnke and Wilmore [4], describing a vertical fold, whereas Lohman, Roche, and Martorell [6] describe a horizontal fold. Given the oblique orientation of the surrounding musculature, an oblique fold may better align with the underlying tissues, making it easier to raise and potentially more comfortable for the participant.

G) *Waist skinfold site*: this skinfold site was included in five documents, four protocols [30,34,37,49], one standard [4], and cited by one protocol [35]. The site is consistently located along the midaxillary line between the 12^th^ rib and the iliac crest, with a vertical fold recommended [4]. However, this orientation differs from the predominantly horizontal and oblique direction of the external oblique muscle fibers.

H) *Abdominal skinfold site*: This was the third most frequently included skinfold site, appearing in 23 documents: 15 protocols [25,29,30,32,34,37,39,41,43–46,49–51], eight standards [3–7,36,48,58], and one additional citation [35]. The standards consistently place the site near the umbilicus, although the specified distance varies: adjacent to the umbilicus [4], 2 cm away [3], 3 cm away [6], or 5 cm away [5,7]. They also differ in fold orientation, with some recommending a vertical fold [3,5,7] and others a horizontal fold [4,6]. Given the vertical orientation of the rectus abdominis muscle, a vertical fold may be easier to raise and more anatomically consistent than a horizontal fold.

I) *Iliac Crest skinfold site*: This skinfold was described in 21 documents, including 14 protocols [27,32,34,37,39–41,43–47,50,51] and seven standards [3–7,48,58]. Although the standards generally place the site near the iliac crest, its precise location varies. Some [4,7] locate it above the iliac crest, others [4,6] at the intersection of the iliac crest and the midaxillary line, whereas Weiner and Lourie [3] define it as 1 cm above and 2 cm medial to the anterior superior iliac spine. Given the anatomical proximity between the iliac crest and supraspinale skinfolds, a site closer to the midaxillary line may provide additional regional information. Fold orientation also differs among standards, with some recommending a vertical fold [3,4] and others a near-horizontal fold [5–7], consistent with the predominantly horizontal orientation of the external oblique muscle fibers in this region. Strictly speaking, however, any angle other than 0° relative to the horizontal axis is oblique, suggesting that the term “near-horizontal” reflects descriptive convention rather than a standardized angular definition.

J) *Supraspinal skinfold site*: This skinfold was described in five documents, including two protocols [42,50] and three standards [5,7,58]. The standards [5,7] consistently define it as a diagonal fold located at the intersection of a horizontal line from the iliac crest and a line extending from the anterior superior iliac spine, following the natural orientation of the external oblique muscle fibers in this region.

K) *Triceps skinfold site*: This was among the most frequently included skinfold sites, appearing in 24 documents: 16 protocols [29,30,32–34,37,39,41–44,46,47,49–51], eight standards [3–7,36,48,58], and four additional citations [27,28,35,40]. The standards [3–7] consistently describe it as a vertical fold on the posterior surface of the arm, in line with the vertical orientation of the underlying muscle fibers. Although all standards position the site at the midpoint of the arm, minor differences exist in how this midpoint is determined.

L) *Biceps skinfold site*: This skinfold was described in nine documents, including four protocols [25,32,40,46], five standards [3,5–7,58], and one additional citation [28]. The standards [5–7] define the biceps skinfold on the anterior surface of the arm at its midpoint, although minor differences exist in how this site is determined. In contrast, Weiner and Lourie [3] locate it above the center of the cubital fossa. Despite these differences, the standards [3,5–7] consistently recommend a vertical fold, aligned with the vertical orientation of the biceps muscle fibers.

M) *Forearm skinfold site*: This was one of the least frequently referenced skinfold sites, appearing in only three documents: one protocol [25] and two standards [3,6]. Although both standards recommend a vertical fold, consistent with the orientation of the underlying muscle fibers, Weiner and Lourie [3] locate the site on the lateral aspect of the forearm at the midpoint of the radius, whereas Lohman, Roche, and Martorell [6] place it on the posterior aspect of the forearm at the point of maximum girth, likely representing a more proximal location.

N) *Thigh skinfold site*: This skinfold site was described in 16 documents, including 10 protocols [25,30,37,39,41,44,45,49–51], six standards [3–7,58], and one additional citation [35]. The standards [3–7] consistently locate the site on the anterior surface of the thigh at the midpoint between the inguinal crease and the superior border of the patella and agree on a vertical fold aligned with the orientation of the quadriceps femoris muscle fibers.

O) *Buttock skinfold site*: This was the least referenced skinfold site, appearing in only one protocol [41]. Sloan [41] located the site on the posterior midline of the thigh above the gluteal fold and described it as a horizontal fold, consistent with the relatively horizontal orientation of the gluteus maximus muscle fibers in this region. Its limited use may reflect its more invasive nature and its potentially lower relevance for representing overall adipose tissue distribution.

P) *Knee skinfold site*: This skinfold site was described in nine documents, including six protocols [29,30,37,45,46,49], three standards [4,6,48], and one additional citation [35]. Both Behnke and Wilmore [4], and Lohman, Roche, and Martorell [6] recommend a vertical fold, consistent with the orientation of the quadriceps femoris muscle fibers. However, they differ slightly in site location, with [4] placing it at the midpoint of the patella and [6] on the anterior surface of the thigh, 2 cm above the proximal border of the patella.

Q) *Calf skinfold site*: This skinfold site was described in 13 documents, including six protocols [25,30,42,46,47,49], seven standards [3–7,48,58], and one additional citation [35]. The standards [3–7] consistently define it as a vertical fold located on the medial aspect of the leg at the level of maximum girth, aligned with the orientation of the gastrocnemius muscle fibers.
